# Effect of Oral Probiotic *Lactobacillus rhamnosus* GR-1 and *Lactobacillus reuteri* RC-14 on the Vaginal Microbiota, Cytokines and Chemokines in Pregnant Women

**DOI:** 10.3390/nu12020368

**Published:** 2020-01-30

**Authors:** Siwen Yang, Gregor Reid, John R.G. Challis, Gregory B. Gloor, Elizabeth Asztalos, Deborah Money, Shannon Seney, Alan D. Bocking

**Affiliations:** 1 Departments of Physiology and Obstetrics and Gynecology, University of Toronto, Toronto, ON M5G 1E2, Canada; siwen.yang@mail.utoronto.ca (S.Y.); lewis.challis@gmail.com (J.R.G.C.); alan.bocking@sinaihealth.ca (A.D.B.); 2 Lunenfeld-Tanenbaum Research Institute, Sinai Health System, Toronto, ON M5G 1X5, Canada; 3 Departments of Microbiology and Immunology, The University of Western Ontario, London, ON N6A 5C1, Canada; 4 Lawson Health Research Institute, 268 Grosvenor Street, London, ON N6A 4V2, Canada; ggloor@uwo.ca (G.B.G.); Shannon.Seney@sjhc.london.on.ca (S.S.); 5 Western Australian Health Translation Network, Perth, WA 6009, Australia; 6 Department of Biochemistry, The University of Western Ontario, London, ON N6A 5C1, Canada; 7 Department of Newborn & Developmental Paediatrics, Sunnybrook Health Sciences Centre, University of Toronto, Toronto, ON M5A 1B2, Canada; elizabeth.asztalos@sunnybrook.ca; 8 Department of Obstetrics and Gynecology, University of British Columbia, 317-2194 Health Sciences Mall, Vancouver, BC V6T 1Z3, Canada; deborah.money@ubc.ca

**Keywords:** probiotics, pregnancy, bacterial vaginosis, microbiota, cytokines, chemokines

## Abstract

Spontaneous preterm birth is associated with vaginal microbial dysbiosis. As certain strains of lactobacilli help restore homeostasis in non-pregnant women, the goal was to determine the effect of *Lactobacillus rhamnosus* GR-1 and *Lactobacillus reuteri* RC-14 administered orally, twice daily for 12 weeks on the vaginal microbiota, cytokines and chemokines of low-risk pregnant women. A double-blind, placebo-controlled, randomized trial comparing probiotic lactobacilli to placebo daily was performed in 86 asymptomatic pregnant women who had an Intermediate or Bacterial Vaginosis Nugent score at 13 weeks. After drop outs, 32 women receiving probiotics and 34 receiving placebo completed the study. The Nugent score returned to normal in 30% of the women in both groups at 28 weeks and was maintained until 35 weeks. The majority of subjects had normal pregnancy outcomes. Ninety-three bacterial species were detected at 13 weeks, with *Lactobacillus iners, Lactobacillus crispatus, Gardnerella vaginalis* and *Atopobium vaginae* being the most abundant across pregnancy. There was no difference in the Shannon diversity index between the probiotic and placebo groups at 13, 28 or 35 weeks. Almost all subjects consumed fermented foods and many of the organisms in the vagina are also known to be present in fermented foods. Interleukin-4 in the placebo group and Interleukin-10 in both probiotic and placebo groups increased slightly at 28 weeks but were not different at 35 weeks when compared to 13 weeks. In conclusion, this study showed no adverse issues resulting from 12 week use of probiotic *Lactobacillus* strains GR-1 and RC-14 during pregnancy in women at low risk for premature birth. The vaginal microbiota demonstrated flux irrespective of this oral probiotic administration.

## 1. Introduction

Healthy human vaginal microbiota, characterized by the dominance of *Lactobacillus* spp., plays an important role in reproductive health and disease. Lactobacilli prevent the overgrowth of pathogens by various mechanisms [1]. Bacterial vaginosis (BV), an altered vaginal microbiota associated with preterm birth (PTB), is characterized by a depletion of lactobacilli and an overgrowth of facultative anaerobic bacteria [2,3,4]. The predominance of pro-inflammatory cytokines over anti-inflammatory cytokines is associated with early onset of labor [5,6]. BV is associated with elevated vaginal concentrations of pro-inflammatory cytokine interleukin (IL)-1β and chemokine IL-8, both of which are also elevated in the amniotic fluid and cervical fluid of women with microbial invasion of the amniotic cavity and preterm delivery [7,8]. 

A Gram stain-based Nugent’s score of 7–10 is widely used to indicate BV [9]. High throughput sequencing techniques to characterize the human vaginal microbiota overcome the inability to grow some microorganisms and the underestimation of vaginal diversity [10,11,12]. Several studies have characterized the vaginal microbiota of healthy pregnant [13,14,15] and non-pregnant women [10,12,16,17] using these methods. 

Probiotics are defined as “live microorganisms which, when administered in adequate amounts, confer a health benefit on the host” [18]. Probiotic lactobacilli can ameliorate BV and replenish lactobacilli in the vagina of non-pregnant women [19,20], and reduce recurrence of urinary tract infections [21,22,23]. The rationale for selecting probiotic *Lactobacillus rhamnosus* GR-1 (GR-1) and *Lactobacillus reuteri* RC-14 (RC-14) was derived from a previous study in non-pregnant women, in which treatment with GR-1 and RC-14 (10^9^ cfu) reduced BV occurrence and recurrence [24]. In addition, GR-1 supernatant possesses anti-inflammatory properties in cultured human intrauterine tissues [25,26,27], mouse macrophages [28] and can reduce inflammation-associated PTB in pregnant mice [29]. We hypothesized that oral administration of GR-1 and RC-14 would present no safety issues and could positively influence the vaginal microbiota, as well as dampen the vaginal concentration of pro-inflammatory cytokines and chemokines. 

## 2. Materials and Methods

### 2.1. Study Participants

Pregnant women with no symptoms of vaginal infections were recruited at Mount Sinai Hospital (MSH), Toronto, Canada. Subjects were over 18 years of age, prior to 17 weeks of gestation, had singleton pregnancies and could provide informed consent. Subjects were excluded if they had multi-fetal pregnancies, fetal complications, a history of previous PTB, second trimester loss, significant maternal medical/surgical complications or HIV. The study was approved by the MSH Research Ethics Board (Approval Number 08-0005-A) following the rules of the Declaration of Helsinki and was registered with ClinicalTrials.gov (Number NCTO1697683). 

As part of the Baseline Eligibility Assessment, information regarding pre-pregnancy weight and height, ethnicity, mode of conception, folic acid intake prior to conception and during the pregnancy, presence or absence of unprotected sex in the previous 4 days, obstetrical history, pre-existing medical conditions, current medications, allergies, smoking, alcohol consumption and illicit drug use during the pregnancy, vaginal and urinary tract infections, antibiotic use during the pregnancy and ingestion of probiotics or fermented foods (yogurt) was obtained. 

Vaginal Swabs were collected under direct visualization using a speculum. Dacron swabs were placed in the posterior fornix or lateral vaginal wall for 10 seconds and then smeared on a glass slide for Nugent scoring. Three additional Dacron swabs were collected using the same technique and stored at -80 for future DNA, cytokine and chemokine analysis. 

### 2.2. Study Groups and Randomization

**Figure 1:**
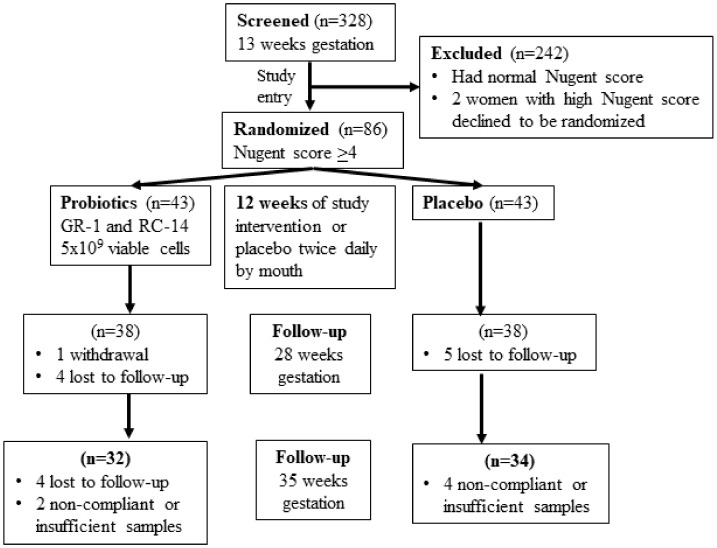
Consort flow chart of pregnant women enrolled in the study.

A total of 328 women between 12- and 16 weeks of gestation consented and were screened between May and October for the presence of an intermediate (4–6) or high (7–10) Nugent score [9]. Eighty-six women whose vaginal samples had a Nugent score ≥ 4 (Figure 1) were randomized. 

A Z test was used to determine the sample size with alpha = 0.05 and power = 0.8, giving a per-group sample size of 40 women to detect a difference between an asymptomatic Intermediate/BV prevalence of 30% in the probiotic group and 60% in the placebo group at the end of treatment protocol. This was increased to 43 in each group to compensate for 5% lost to follow-up. Subjects were randomized using a web-based service to receive two identical looking capsules per day containing either GR-1 and RC-14 or placebo given orally for 12 weeks. Vaginal swabs were collected at 13-, 28- and 35 weeks of gestation and analyzed for Nugent score, cytokines, chemokines and microbiota. Fourteen subjects were lost to follow-up or withdrew, 3 had taken less than 25% of the capsules, and there was insufficient sample for analysis in 3 others. This left 32 subjects in the probiotic and 34 in the placebo group with samples available for sequencing analysis and samples from 31 probiotic and 33 placebo subjects for cytokines and chemokine analysis. 

### 2.3. Nugent Score

Vaginal swab smears were graded on a 10-point scale based on the presence or absence of various bacterial morphotypes, including *Lactobacillus* spp., pathogenic *Gardnerella vaginalis* and *Bacteroides* spp. A score of 0–3 represents a normal vaginal microbiota, with high abundance of *Lactobacillus* spp., a score of 4–6 represents an Intermediate biota with higher proportions of non-*Lactobacillus* morphotypes and a score of 7–10 represents BV, with depleted lactobacilli and a high abundance of pathogenic morphotypes [9]. 

### 2.4. Probiotic Strains

*Lactobacillus rhamnosus* GR-1 and *Lactobacillus reuteri* RC-14 and placebo capsules (powder without the organisms) were provided by Chr Hansen, Denmark. The freeze-dried organisms (2.5 × 10^9^ of GR-1 and 2.5 × 10^9^ RC-14) were in gelatin capsules with a total of 180 mg of powder, including anhydrous dextrose and potato starch fillers, microcrystalline cellulose binder and magnesium stearate lubricant. 

### 2.5. DNA Isolation and Polymerase Chain Reaction (PCR) Amplification of V6 Region of 16S rDNA

Vaginal swabs were equilibrated in 800μL phosphate buffer saline (PBS) on ice and vortexed for 1 min. DNA was extracted with a Qiagen Stool Extraction Kit (Qiagen, Toronto, Canada), bacterial DNA was amplified with barcoded primers targeting the V6 region of the 16S rDNA and PCR amplification was performed with colorless GO-Taq hot start master mix (Promega, Canada) for 25 repeating cycles of 95 °C, 55 °C and 72 °C for 1 minute each step. The amplified products were quantified using a QuBit broad-range double-stranded DNA fluorometric quantitation reagent kit (Life technologies, Canada). Samples were pooled at equal molar concentrations and purified using Wizard PCR Clean-Up Kits (Promega, Canada).

### 2.6. Sequencing

Barcoded DNA was sequenced in pairs on a MiSeq Illumina platform. V6L and V6R primers included a unique 12bp sequence tag to barcode each sample. The primers used were: V6L-5′- *ACACTCTTTCCCTACACGACGCTCTTCCGATCT*N (12) CWACGC.

GARGAACCTTACC-3′ and V6R-5′-CGGTCTCGGCATTCCTGCTGAACCGCTCTTCCG.

*ATCN (12)* ACRACACGAGCTGACGAC-3′, where the sequences prior to N (12) are the Illumina MiSeq sequencing primers and the residues after denote the universal 16S rRNA gene primers. The N (12) indicates 4 random nucleotides followed by 8mer barcodes, as described previously [4]. The sequence results were provided in fastq format. All sequences were filtered to remove reads with indeterminate bases, overlapped, and a table of counts was generated for each sample containing sequences grouped at 97% operational taxonomic unit (out) and 100% identical sequence unit identity, as described previously [10]. The sequences were then classified to distinct taxonomic species using the online Ribosomal Database Project (http://rdp.cme.msu.edu/seqmatch/seqmatch_intro.jsp). Sequences not identical across all best matches were marked as unclassified. 

### 2.7. Protein Extraction and Cytokine/Chemokine Multiplex Assay

Vaginal swab samples were equilibrated in Tris-HCl buffer (pH 7.5) with 150 mmol/L NaCl, 1 mmol/L phenylmethylsulfonyl fluoride (Sigma), 0.05% Tween-20 (Sigma) and a protease inhibitor cocktail tablet (Roche) for 30 min at 4 °C and vortexed every 10 min. The swab was removed, and buffer was centrifuged at 16,000× *g* for 15 min at 4 °C. Supernatant was stored at −80 °C in aliquots until further analysis. IL-1 receptor antagonist (IL-1rα), IL-1β, IL-2, IL-4, IL-5, IL-6, IL-7, IL-8, IL-9, IL-10, IL-12p70, IL-13, IL-15, IL-17, basic Fibroblast Growth Factor (bFGF), Colony Stimulating Factor (CSF) 2, CSF3, Interferon (IFN)-γ, CXCL10, CCL2, CCL3, CCL4, CCL5, CCL11, Platelet-Derived Growth Factor (PDGF)-bb, Tumor Necrosis Factor (TNF)-α and Vascular Endothelial Growth Factor (VEGF) were measured with a 27 human multiplex cytokine/chemokine kit (Biorad, Mississauga, Canada). 

### 2.8. Statistical Analyses

Statistical Analysis employed Unpaired Student’s t-tests (two tailed), Chi-square test and Two-Way Repeated Measure Analysis of Variance (ANOVA) followed by the Holm–Sidak method (SigmaStat, version 3.5) where appropriate. Sequencing data were analyzed using a compositional data analysis paradigm [30], coupled with Bayesian estimation of background frequencies. Briefly, read counts per OTU were treated as probabilities of observation conditioned on the total sample read count [31,32]. The background frequency was modeled as coming from a Dirichlet distribution and the centered ratio logarithm transformation was performed on the distribution [33,34]. Statistical analysis of the sequencing data was carried out with the ALDEx2 package from Bioconductor using R (version 3.0.1). The Generalized Estimation Equation Model was used for data that did not follow the normal distribution. Data were adjusted for false discovery rate using the Benjamini–Hochberg procedure and an adjusted *p*-value of *p* < 0.05 was considered statistically significant. Data were tested for normality and equal variance and were expressed as mean values ± standard deviation (SD). The Shannon diversity index was calculated using standard protocols [35]. 

## 3. Results

### 3.1. Pre-Randomization Characteristics

As determined at 13 weeks, the mean age of subjects was 33.8 ± 4.2 years and the mean pre-pregnancy body mass index (BMI) was 22.5 ± 3.2 for the probiotic group, which were not different from the placebo group (age of 34.4 ± 3.3 years and BMI of 22.4 ± 3.1) (Table 1). Over 55% of the women were Caucasian in both groups. Other ethnicities included South and East Asian, Black and Hispanic. 

Forty of the 43 women (93%) in both the placebo and the probiotic groups had a natural conception (Table 1). Seventeen pre-existing conditions were reported in 14 women in the probiotic group and 27 pre-existing conditions in 21 women in the placebo group. The majority of women (81.4% probiotic group; 95.4% placebo) reported ingesting probiotic-containing food products (fermented foods) during pregnancy (*p* > 0.05, Table 1).

### 3.2. Pregnancy Outcomes

Pregnancy outcome data were available for 41 subjects in the probiotic group and 43 in the placebo group. Antibiotics were taken by 14.6% of subjects in the probiotic group and 11.6% in the placebo group for various indications (*p* > 0.05, Table 2). There was no significant difference in antibiotic administration during labor (46.3% probiotic group, 37.2% placebo group). Labor was induced in 19.5% of subjects in the probiotic group and 80.5% had a vaginal delivery, similar to the placebo group. There was no difference in the rate of Caesarian section between the two groups. Six of the eight women in the probiotic group underwent Caesarian section in labor (emergency), whereas 7 of the 9 women underwent elective (pre-labor) Caesarian section. This was not statistically different. The mean gestational age at delivery was the same in both groups (39.1 ± 1.4 weeks in the probiotic group, 39.4 ± 0.9 weeks in placebo, Table 2). There was no difference in mean birth weight (3340 ± 433.7 grams, probiotic; 3351 ± 463.5 grams, placebo). One infant in the placebo group had intrauterine growth restriction (IUGR) based on published growth curves for Canadian newborns [36], and two infants in the probiotic group were delivered at 34 weeks of gestation in association with preterm premature rupture of membranes (PPROM). There was no difference in the fetal sex distribution or cord blood pH between the two groups. No adverse reactions were reported with either probiotic or placebo intake. 

### 3.3. Compliance

After treatment, there were on average 13 pills (7.7%) left in the bottles returned by subjects in the probiotic group and 9 pills (5.4%) in the placebo group. Twenty-six of the 32 subjects in the probiotic group (81.2 %) and 29 of the 34 in the placebo group (85.3%) had taken over 75% of the total pills (168 pills) (*p* > 0.05). The remaining women had taken over 50% of the total pills. Three women had taken less than 25% of the total pills and were excluded from the analyses. 

### 3.4. Vaginal Microbiota

One aim of this trial was to determine if oral probiotic intake changed the vaginal microbiota, assessed by Nugent Gram stain scoring and high throughput sequencing. There were 11 out of 32 women (34.4%) in the probiotic group and 11 out of 34 (32.3%) in the placebo group, with normal Nugent score at both 28 weeks (*p* > 0.05) and 35 weeks (*p* > 0.05). 

**Figure 2:**
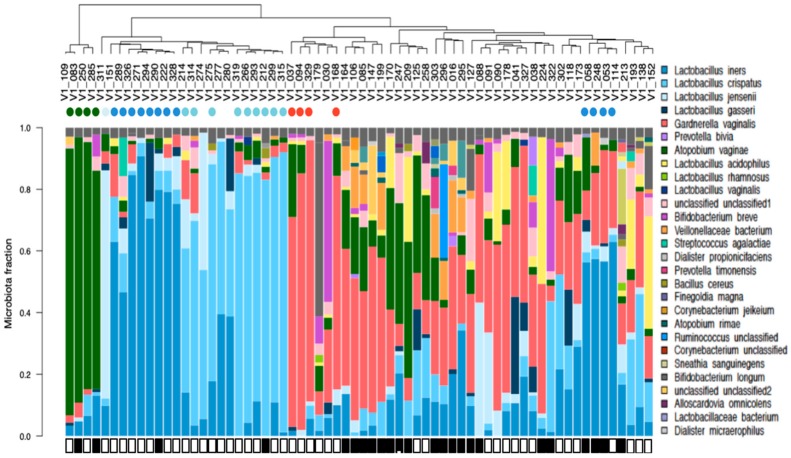
Vaginal microbiota clustered by bacterial similarity in pregnant women prior to treatment, at 13 weeks of gestation (n = 66). Each bar represents the vaginal microbiota of a single woman and corresponds to the participant identification (ID) number labeled in the dendogram, clustered using average linkage cluster analysis. Species found in >1% abundance are represented by a unique color. Species with <1% abundance in the sample are pooled into a single fraction at the top of the bar in grey color. Women who have a single bacterial species which dominated more than 40% of their vaginal microbiota are identified with a color dot below their identification number that corresponds to the dominant species (*Dark green, Atopobium vaginae, n* = 4; *Very light blue, Lactobacillus (L.) jensenii, n* = 1; *blue, L. iners, n* = 12; *light blue, L. crispatus, n* = 9 and red, *Gardnerella vaginalis, n* = 4). Black rectangles are used to denote women with a bacterial vaginosis (BV) Nugent score, and white rectangles are used to identify women with an Intermediate Nugent score.

**Figure 3:**
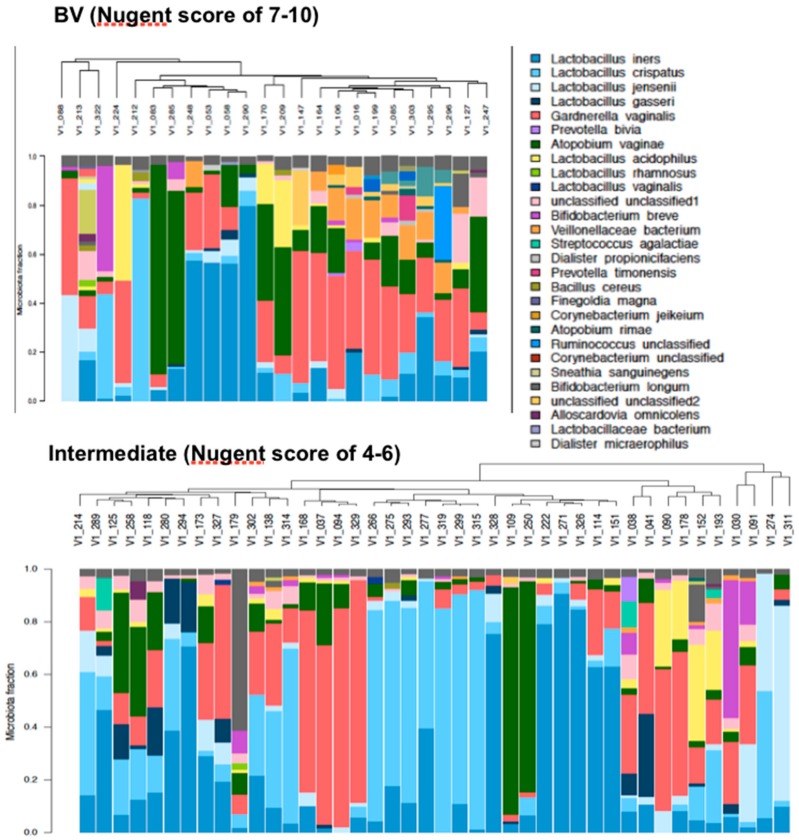
Vaginal microbiota clustered by bacteria similarity in pregnant women with a BV (*n* = 24) or an Intermediate (*n* = 42) Nugent score prior to treatment, at 13 weeks of gestation. Each bar represents the vaginal microbiota of one woman and corresponds to the identification number labeled in the dendogram, clustered using average linkage cluster analysis. A unique color is used to represent species found in >1% abundance. Species with <1% abundance are pooled into a fraction at the top in grey color.

Ninety-three bacterial species were detected by sequencing at 13 weeks with *Lactobacillus iners, Lactobacillus crispatus, Gardnerella vaginalis* and *Atopobium vaginae* being the most abundant across pregnancy. Thirty of sixty-six women had a single dominant (>40% of their microbiota) bacterial species (*A. vaginae, n* = 4; *L. jensenii, n* = 1; *L. iners, n* = 12, *L. crispatus, n* = 9 and *G. vaginalis, n* = 4) at 13 weeks (Figure 2). The microbiota of pregnant women with an intermediate Nugent score (*n* = 42) and those with a BV Nugent score (*n* = 24) at the time of study entry were not different between these groups (*p* > 0.5) and their results were pooled for all subsequent analyses (Figure 3). 

**Figure 4:**
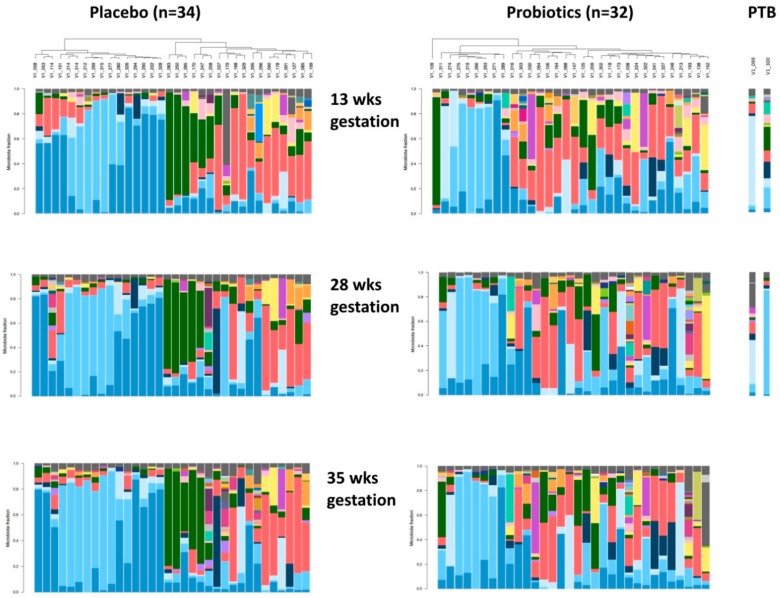
Vaginal microbiota across pregnancy clustered by bacteria similarity in pregnant women who received either placebo (*n* = 34) or probiotic (*n* = 32) treatment. Y Axis = Microbiota Fraction. Each bar represents the vaginal microbiota of a single woman and corresponds to the identification number labeled in the dendogram, clustered using average linkage cluster analysis. Species found in >1% abundance are represented by a unique color and species that have <1% abundance are pooled into a single fraction at the top of the bar in grey color. Women were aligned in the same vertical column at 13, 28 and 35 weeks of gestation. Women who have undergone preterm birth (PTB) (*n* = 2) in the probiotic group are denoted with white squares. There was no effect due to BV or Intermediate status.

The microbiota of subjects who received placebo was similar to those receiving probiotics at the end of the 12-week treatment and also at 35 weeks gestation (Figure 4, Table 3 and Table 4). 

**Figure 5:**
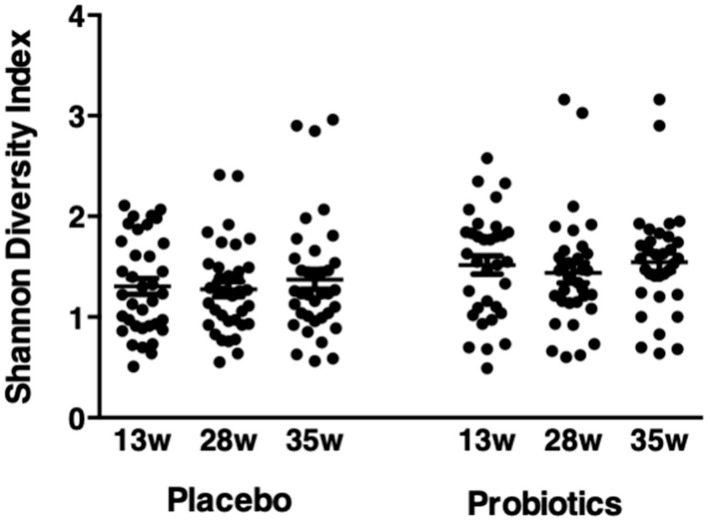
Shannon diversity index (SDI) across gestation in pregnant women who received either placebo or probiotic treatment. Results are mean values ± standard deviation (SD) and are expressed in ratios. Comparisons between the probiotic (n = 32) and placebo (n = 34) groups at 13, 28 and 35 weeks of gestation were assessed with Two-Way Repeated Measure Analysis of Variance (ANOVA) followed by the Holm–Sidak post hoc test (*p* > 0.05).

There was no difference in the microbiota between the placebo and probiotic groups when data were grouped by ethnicity, pre-pregnancy BMI or when women whose microbiota were dominated by *Lactobacillus* spp. were excluded. The relative mean abundance of 12 species including *L. iners, L. acidophilus*, *G. vaginalis* and *A. vaginae* decreased at 28 weeks and/or 35 weeks of gestation in the placebo group and/or the probiotic group, compared to 13 weeks of gestation (Table 3). In contrast, the relative mean abundance of nine species increased across pregnancy (Table 4). There was no difference in the Shannon diversity index between the probiotic and placebo groups at 13, 28 or 35 weeks (Figure 5). 

*Lactobacillus rhamnosus* was detected in the vagina of 98% of the women (65 out of 66 women) at 13 weeks of gestation, and its abundance did not change with probiotic treatment. Two women in the probiotic group delivered at 34 weeks of gestation in association with PPROM. In one, the vaginal microbiota was dominated by *L. jensenii,* and following probiotic treatment, her biota became more heterogeneous, with increased abundance of *L. gasseri*, *G. vaginalis* and *Prevotella bivia*. The other woman initially had a heterogenous vaginal microbiota, and with probiotic treatment, *L. crispatus* dominated her microbiota (Figure 4).

### 3.5. Cytokines/Chemokines

The concentrations of cytokines and chemokines at the time of study entry were not different between pregnant women diagnosed with an Intermediate or BV Nugent score. Therefore, these data were combined in subsequent analyses. The concentration of cytokines and chemokines were not different between placebo (*n* = 33) and probiotic-treated (*n* = 31) women at 13, 28 or 35 weeks of gestation (*p* > 0.05, Table 5). 

**Figure 6:**
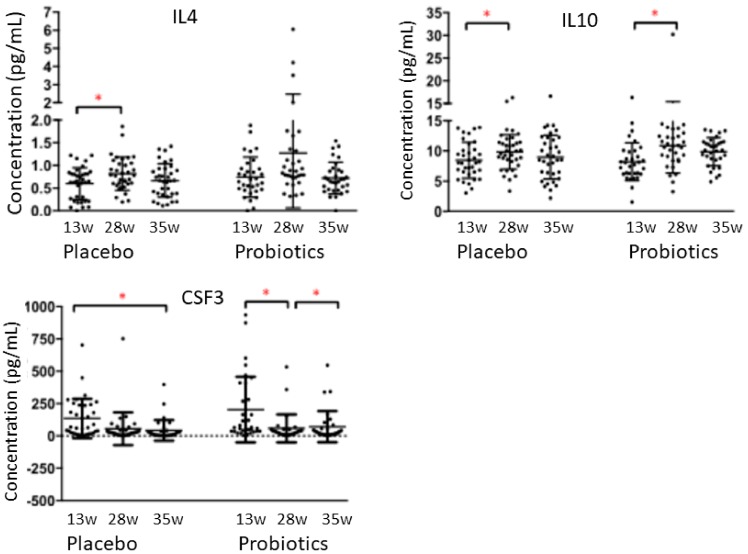
Concentrations of cervico-vaginal cytokines IL-4, IL-10 and CSF3 across gestation in pregnant women who received either placebo or probiotic treatment. Results are mean values ± SD and are expressed in picogram per milliliter. Comparison between the placebo group (*n* = 33) and the probiotic group (*n* = 31) was assessed with the Generalized Estimation Equation model in R. * = *p* < 0.05.

Overall, the cervico-vaginal cytokines and chemokines showed considerable variation across the collection times. The concentrations of the anti-inflammatory cytokines IL-4 in the placebo group and IL-10 in both probiotic and placebo groups increased slightly at 28 weeks of gestation, but were not different at 35 weeks of gestation, when compared to 13 weeks (*p* < 0.05, Figure 6). CSF3 decreased at 28 weeks in the probiotic group and at 35 weeks of gestation in the placebo group, when compared to 13 weeks (*p* < 0.05, Figure 6). Levels of the remaining pro-inflammatory cytokines, anti-inflammatory cytokines, chemokines and growth/hematopoietic factors did not change throughout pregnancy (*p* > 0.05, Table 5). Concentrations of IL-2, IL-5, IL-15 and IL-1ra were outside the detection limit.

## 4. Discussion

This study showed that 12-week oral administration of probiotic *Lactobacillus* strains GR-1 and RC-14 is safe for use during low-risk pregnancy. The rate of preterm labor was too low to determine whether probiotic therapy had an impact, similar to a previous large study [37]. 

The Nugent scoring system indicated that the subjects had either BV or an Intermediate status, yet lactobacilli dominated the vagina in more than one third of them at 13 weeks of gestation. This indicates the unreliability of the Nugent scoring system for diagnosis [4]. A DNA level of ≥10^9^ copies/mL for *G. vaginalis* and ≥10^8^ copies/mL for *A. vaginae* has a 95% sensitivity and positive predictive value, and 99% specificity and negative predictive value for the diagnosis of BV, much higher than the Nugent score [38]. Of note, there was no difference in pregnancy outcomes between women who had an Intermediate Nugent score at 13 weeks when compared to those with BV.

The probiotic dosage was based upon studies in non-pregnant women [24]. We now know that pregnant women have a higher abundance of *Lactobacillus* spp. including *L. crispatus, L. gasseri* and *L. jensenii,* and a more resilient microbiota than non-pregnant women [13,15]. Thus, the finding that the three months of probiotic therapy did not alter the microbiota may reflect too low a dose, or an inability to displace indigenous lactobacilli [39,40]. Of note, as metabolomics were not performed, we cannot state whether the microbiota function was altered or not as a result of the probiotic administration. 

The high prevalence of *L. rhamnosus* as a vaginal commensal was unexpected as this species is not one of the five (*L. crispatus, L. iners, L. gasseri, L. jensenii* and *L. reuteri or L. vaginalis*, depending on the country) detected in most microbiota studies of healthy women [41,42,43]. This may have been a result of the subjects ingesting fermented foods during the study. Presence of these strains may have been a barrier to *L. rhamnosus* GR-1 persistence given the point made above that probiotic lactobacilli do not persist if the same species is already present in the indigenous microbiota [39]. 

An interesting finding was the high incidence of consumption of fermented foods. This led to the suggestion that it may have resulted in more *L. rhamnosus* being present in the vagina. Many of the genera identified can be found in fermented foods [44,45,46,47], raising the possibility that consumption of these foods may provide bacteria that are beneficial to vaginal health during pregnancy. 

The elasticity of the vaginal microbiota, in agreement with a previous study [14], was observed by a decline in *A. vaginae*, *A. rimae* and *G. vaginalis*. In contrast to studies that target the V1-V3 [14] and V3-V4 [12,48] regions of the 16S rDNA, we did not observe a change in the relative abundance of *Gemella* and *Sneathia sanguinegens*, nor did we detect *Eggerthella* spp., *Parvimonas micra*, BV-associated bacteria 1 (BVAB1), BVAB2 or *Ureaplasma parvum*. The use of primers that targeted the V6 region in this study may have under-estimated the presence of these bacteria [10,11]. Using sequencing primers that target the cpn60 gene, it is possible to measure the abundance of Mollicutes in non-pregnant women [16] as well as in pregnant women, during which the prevalence of Mollicutes appears to be lower [49]. The relative abundance of *L. iners and L. acidophilus* across gestation decreased in this study, in contrast to a report finding an increase in several *Lactobacillus* spp. (*L. crispatus*, *L. jensenii*, *L. gasseri* and *L. vaginalis)* with advancing gestational age in healthy women [14]. The absence of *Sneathia amnii* in both groups was notable given the recent suggestion that it is associated with preterm birth [50].

Previous studies in term-cultured human intra-uterine tissues and in pregnant mice, demonstrated that *L. rhamnosus* GR-1 supernatant possesses anti-inflammatory properties [25,26,27,29]. As the current subjects did not have an underlying state of inflammation, no anti-inflammatory induction was necessary. The use of probiotics administered intravaginally, including the GR-1 and RC-14 strains, may be more likely to influence the microbiota as well as the inflammatory status [51,52]. Nevertheless, there was a shift towards an anti-inflammatory environment across gestation, as was made evident by an increase in vaginal IL-4 and IL-10 concentrations at 28 weeks, which were in a comparable range with previous studies [53,54]. CSF3, which is important in placentation and neutrophil progenitor proliferation, differentiation and survival, decreased with advancing gestational age. CSF3 possesses anti-inflammatory properties in cultured human placental trophoblast cells [26]. However, elevated maternal CSF3 concentrations have also been associated with spontaneous PTB in humans [55]. 

In conclusion, this study showed that oral probiotic treatment with probiotic GR-1 and RC-14 did not result in adverse outcomes, in agreement with other studies [37,56]. The characteristics of *Lactobacillus rhamnosus* GR-1 determined in vitro that predicted an ability to alter the vaginal environment of pregnant women via oral administration, did not translate to humans when the organism was administered orally with *L. reuteri* RC-14. That said, many women delivered normally which suggests that the probiotic was not essential for health in women consuming fermented foods, and the cohort was not devoid of lactobacilli or at risk of preterm labor. Future studies of probiotics should include women at risk of preterm delivery, administer a higher lactobacilli oral dose or use intravaginal instillation and more closely examine fermented food products and regularity of consumption. 

## Figures and Tables

**Table 1:** Characteristics of pregnant women randomized at 13 weeks of gestation. Comparison between the probiotic group and the placebo group was performed with Student’s t-test or Chi-square (*p* > 0.05).

Baseline Characteristic	Probiotic Group *n* = 43 (%)	Placebo Group *n* = 43 (%)
Maternal Age (years)	33.8 ± 4.2	34.4 ± 3.3
Pre-pregnancy Body Mass Index	22.5 ± 3.2	22.4 ± 3.1
Week of vaginal swab collection	13.2 ± 1.1	13.3 ± 1.1
Ethnicity *		
Caucasian	18 (56.3)	22 (64.8)
South Asian	2 (6.2)	3 (8.8)
East Asian	6 (18.8)	7 (20.6)
Black	2 (6.2)	0
Hispanic	2 (6.2)	2 (6.2)
Mixed	1 (3.1)	0
Other	1 (3.1)	0
Mode of conception		
Natural	40 (93)	40 (93)
Assisted	3 (7)	3 (7)
Ovulation induction	1	1
IVF	0	1
Intracystoplasmic sperm injection	0	1
Donor sperm	1	0
IUI	1	0
Pre-existing medical conditions	17 (39.5)	27 (62.8)
Depression/anxiety disorder	1	9
Endocrine disorders	5	3
Hematological	1	3
Musculoskeletal	2	3
Gastrointestinal	0	3
Cardiovascular	2	0
Genitourinary/Urinary/Gynecological	4	1
Respiratory	0	4
Others	2	1
Previous surgeries in past 10 years	16 (37.2)	34 (79)
Obstetrical/gynecological	12	23
Others	4	11
Current medications	16 (37.2)	22 (51.2)
Anti-nauseants	1	3
Vitamin D	1	1
Antidepressants	2	4
Natural supplements	5	2
Thyroid medications	4	1
Analgesics	0	1
Iron supplements	1	3
Gastrointestinal medications	2	1
Asthma medications	0	5
Others	0	1
Fermented food ingested during pregnancy	35 (81.4)	41 (95.4)
Natural supplement	2 (4.7)	2 (4.7)

* Ethnicity is based on 32 women in the placebo group and 34 women in the probiotic group. IVF—In vitro fertilization; IUI—Intrauterine Insemination.

**Table 2:** Comparison of pregnancy outcomes between the probiotic group (*n* = 41) and the placebo group (*n* = 43) was performed with Student’s t-test or Chi-square (*p* > 0.05).

Labour and Delivery Characteristics	Probiotic Group *n* = 41 (%)	Placebo Group *n* = 43 (%)
Antibiotics during pregnancy *	6 (14.6)	5 (11.6)
Antibiotics during labor and delivery	19 (46.3)	16 (37.2)
Induction of labor	8 (19.5)	9 (20.9)
Mode of Delivery		
Vaginal	33 (80.5)	34 (79.1)
Spontaneous	28/33 (84.9)	28/34 (82.4)
Assisted	5/33 (15.2)	6/34 (17.7)
C-section	8 (19.5)	9 (20.9)
Emergency	6/8 (75.0)	2/9 (22.2)
Elective	2/8 (25.0)	7/9 (77.8)
Gestational age at delivery (weeks)	39.1 ± 1.4	39.4 ± 0.9
Birth weight (g)	3340 ± 433.7	3351 ± 463.5
IUGR (<3rd percentile)	0	1 (2.3)
Preterm birth (<37 weeks of gestation)	2 (4.9)	0
Apgar score <7 at 5 minutes	1 (2.4)	0
Fetal Sex		
Male	19 (46.3)	24 (55.8)
Female	22 (53.7)	19 (44.2)
Cord blood pH	7.26 ± 0.07	7.26 ± 0.08

* Antibiotics included penicillin, teva-cloxacillin, erythromycin, amoxicillin, macrobid, clindamycin, biaxin, ciprofloxacin, cephalexin and topical metronidazole. IUGR – intrauterine growth restriction

**Table 3:** The relative to mean abundance of vaginal bacteria species that decreased across gestation in pregnant women treated with placebo or probiotics.

	Placebo Group			Probiotic Group			
Species	13 Weeks	28 Weeks	35 Weeks	13 Weeks	28 Weeks	35 Weeks	*p*-Value
*Lactobacillus iners*	10.7 ± 2.7 ^a’^	10.5 ± 2.5 ^a’^	10.1 ± 2.5 ^b’^	9.8 ± 2.1 ^a^	9.8 ± 2.5 ^a^	9.5 ± 2.4 ^a^	0.006
*Gardnerella vaginalis*	10.3 ± 2.4 ^a’^	10.0 ± 2.2 ^a’^	9.3 ± 2.4 ^b’^	10.5 ± 2.4 ^a^	9.9 ± 2.4 ^b^	9.6 ± 2.5 ^b^	<0.002
*Atopobium vaginae*	9.4 ± 2.2 ^a’^	9.3 ± 2.2 ^a’^	8.8 ± 2.2 ^b’^	9.0 ± 2.3 ^a^	8.6 ± 2.4 ^b^	8.7 ± 2.4 ^a,b^	0.002
*Lactobacillus acidophilus*	6.6 ± 2.6 ^a’^	5.7 ± 2.7 ^b’^	5.3 ± 2.7 ^c’^	6.8 ± 2.5 ^a^	6.4 ± 2.4 ^a^	6.4 ± 2.4 ^a^	<0.006
*Atopobium rimae*	2.8 ± 2.2 ^a’^	2.3 ± 2.3 ^a’^	1.6 ± 2.3 ^b’^	2.5 ± 2.3 ^a^	1.9 ± 2.0 ^b^	1.7 ± 2.6 ^b^	<0.002
*Bacillus cereus*	2.8 ± 2.5 ^a’^	1.6 ± 2.4 ^b’c’^	1.9 ± 2.2 ^c’^	2.5 ± 2.9 ^a^	1.5 ± 2.9 ^b^	1.7 ± 2.9 ^b^	<0.001
*Lactobacillaceae bacterium*	1.6 ± 3.2 ^a’^	−0.7 ± 1.4 ^b’^	−1.3 ± 1.5 ^c’^	−0.2 ± 2.5 ^a^	−1.2 ± 1.6 ^b^	−1.4 ± 1.2 ^b^	<0.008
*Escherichia coli*	1.2 ± 1.8 ^a’^	−0.7 ± 1.7 ^b’^	−0.2 ± 2.1 ^b’^	1.3 ± 2.5 ^a^	−0.4 ± 2.1 ^b^	0.0 ± 1.9 ^b^	<0.002
*Desulfotomaculum halophilum*	1.4 ± 2.6 ^a’^	0.4 ± 2.6 ^b’^	−0.8 ± 2.4 ^c’^	0.5 ± 2.5 ^a^	−0.9 ± 2.1 ^b^	−1.1 ± 2.3 ^b^	<0.002
*Streptococcus thermophiles*	−0.3 ± 2.3 ^a’^	−2.4 ± 1.3 ^b’^	−2.4 ± 1.6 ^b’^	0.2 ± 2.3 ^a^	−1.7 ± 1.6 ^a^	−2.3 ± 1.6 ^a^	<0.006
*Erythrobacter flavus*	−1.7 ± 2.4 ^a’^	−3.0 ± 1.1 ^b’^	−3.1 ± 1.4 ^b’^	−1.4 ± 2.6 ^a^	−2.6 ± 1.7 ^b^	−2.9 ± 1.6 ^b^	<0.006
*Prevotella denticola*	−2.5 ± 0.8 ^a’^	−2.8 ± 1.8 ^a’^	−3.4 ± 1.0 ^b’^	−2.2 ± 1.0 ^a^	−2.7 ± 1.2 ^a,b^	−3.1 ± 1.1 ^b^	<0.003

Results are mean values ± SD and are expressed in centered logarithm transformed ratios. Comparisons between the placebo (*n* = 34) and probiotic (*n* = 32) groups at 13, 28 and 35 weeks of gestation were assessed using the Generalized Estimation Equation model in R. Statistical significance within the placebo group (a’, b’ and c’) and within the probiotic group (a, b and c) is denoted with different letters (*p* < 0.05).

**Table 4:** The relative to mean abundance of vaginal bacterial species that increased across gestation in pregnant women treated with placebo or probiotics.

	Placebo Group			Probiotic Group			
Species	13 Weeks	28 Weeks	35 Weeks	13 Weeks	28 Weeks	35 Weeks	*p*-Value
*Corynebacterium pseudogenitalium*	−0.9 ± 1.5 ^a’^	0.4 ± 1.6 ^b’^	1.0 ± 1.8 ^c’^	−1.1 ± 1.8 ^a^	1.0 ± 2.4 ^b^	1.0 ± 1.7 ^b^	<0.002
*Facklamia hominis*	−1.6 ± 1.0 ^a’^	−1.4 ± 1.4 ^a’^	−0.8 ± 1.8 ^b’^	−1.8 ± 1.1 ^a^	−0.9 ± 1.6 ^b^	−1.0 ± 1.6 ^b^	<0.004
*Corynebacterium amycolatum*	−1.9 ± 0.9 ^a’^	−1.1 ± 1.5 ^b’^	−0.5 ± 1.8 ^c’^	−1.6 ± 1.0 ^a^	−0.7 ± 1.7 ^b^	−0.3 ± 1.1 ^b^	<0.008
*Clostridiales coagulans*	−1.8 ± 0.9 ^a’^	−1.0 ± 1.7 ^b’^	−0.3 ± 2.0 ^c’^	−1.9 ± 1.0 ^a^	−1.3 ± 1.8 ^b^	−1.3 ± 1.7 ^b^	<0.003
*Varibaculum cambriense*	−1.7 ± 1.0 ^a’^	−0.4 ± 1.8 ^b’^	0.0 ± 1.7 ^b’^	−1.6 ± 1.3 ^a^	−0.7 ± 1.5 ^b^	−0.5 ± 1.8 ^b^	<0.005
*Campylobacter ureolyticus*	−2.0 ± 0.9 ^a’^	−1.8 ± 1.7 ^b’^	−1.1 ± 1.5 ^b’^	−1.6 ± 1.6 ^a^	−1.6 ± 1.6 ^a^	−1.4 ± 2.1 ^b^	<0.004
*Corynebacterium coyleae*	−2.2 ± 1.4 ^a’^	−1.1 ± 2.4 ^b’^	−1.4 ± 2.4 ^b’^	−2.1 ± 1.1 ^a^	−1.7 ± 1.6 ^b^	−1.7 ± 1.5 ^b^	<0.002
*Prevotella disiens*	−0.9 ± 1.5 ^a’^	0.4 ± 1.6 ^b’^	1.0 ± 1.8 ^b’^	−1.1 ± 1.8 ^a^	1.0 ± 2.4 ^a^	1.0 ± 1.7 ^a^	<0.003
*Cryptobacterium curtum*	−1.6 ± 1.0 ^a’^	−1.4 ± 1.4 ^a’^	−0.8 ± 1.8 ^b’^	−1.8 ± 1.1 ^a^	−0.9 ± 1.6 ^a^	−1.0 ± 1.6 ^b^	<0.005

Results are mean values ± SD and are expressed in centered logarithm transformed ratios. Comparisons between the placebo (*n* = 34) and probiotic (*n* = 32) groups at 13, 28 and 35 weeks of gestation were assessed using the Generalized Estimation Equation model in R. Statistical significance within the probiotic group (a’, b’ and c’) and within the placebo group (a, b and c) is denoted with different letters (*p* < 0.05).

**Table 5:** Cervico-vaginal cytokines and chemokines across gestation in pregnant women who received either placebo or probiotic treatment.

	Placebo Group (*n* = 33)			Probiotic Group (*n* = 31)		
	13 Weeks	28 Weeks	35 Weeks	13 Weeks	28 Weeks	35 Weeks
IL·1J3	121.3 ± 186.6 ^a’^	80.7 ± 171.6 ^a’^	72.2 ± 166.8 ^a’^	199.7 ± 404.2 ^a^	66.4 ± 143.3 ^a^	82.3 ± 113.2 ^a^
IL-2	0.6 ± 0.7 ^a’^	0.6 ± 0.6 ^a’^	0.4 ± 0.5 ^a’^	0.4 ± 0.6 ^a^	1.2 ± 2.8 ^a^	0.6 ± 0.6 ^a^
IL-4	0.6 ± 0.4 ^a’^	0.8 ± 0.4 ^b’^	0.7 ± 0.4 ^a’,b’^	0.8 ± 0.4 ^a^	1.3 ± 1.2 ^a^	0.7 ± 0.4 ^a^
IL-5	0.3 ± 0.3 ^a’^	0.4 ± 0.5 ^a’^	0.4 ± 0.3 ^a’^	0.6 ± 1.2 ^a^	1.1 ± 2.1 ^a^	0.5 ± 0.3 ^a^
IL-6	15.1 ± 30.5 ^a’^	4.1 ± 5.6 ^a’^	3.4 ± 5.0 ^a’^	36.0 ± 71.1 ^a^	6.3 ± 10.7 ^a^	5.3 ± 8.0 ^a^
IL-7	56.0 ± 121.4 ^a’^	34.6 ± 33.7 ^a’^	29.9 ± 37.1 ^a’^	55.4 ± 117.5 ^a^	90.4 ± 269.4 ^a^	29.3 ± 36.2 ^a^
IL-8	1453.9 ± 2230.1 ^a’^	1155.8 ± 2716.0 ^a’^	604.4 ± 985.1 ^a’^	2068.0 ± 4658.2 ^a^	418.2 ± 442.6 ^a^	855.0 ± 1258.5 ^a^
IL-9	7.9 ± 16.2 ^a’^	4.6 ± 4.1 ^a’^	4.5 ± 6.7 ^a’^	6.4 ± 12.1 ^a^	16.5 ± 57.3 ^a^	3.8 ± 3.8 ^a^
IL-10	8.4 ± 2.9 ^a’^	10.0 ± 2.7 ^b’^	9.0 ± 3.6 ^a’,b’^	8.4 ± 3.2 ^a^	11.0 ± 4.6 ^b^	9.9 ± 2.4 ^a,b^
IL-12p70	57.8 ± 89.8 ^a’^	55.0 ± 47.0 ^a’^	44.6 ± 38.1 ^a’^	50.3 ± 72.7 ^a^	90.0 ± 217.5 ^a^	41.4 ± 22.1 ^a^
IL-13	4.7 ± 8.8 ^a’^	3.4 ± 2.1 ^a’^	3.3 ± 3.0 ^a’^	5.1 ± 9.8 ^a^	9.7 ± 27.8 ^a^	3.1 ± 2.8 ^a^
IL-15	1.0 ± 1.1 ^a’^	1.3 ± 1.5 ^a’^	0.8 ± 0.9 ^a’^	1.0 ± 1.0 ^a^	1.6 ± 1.6 ^a^	0.8 ± 0.9 ^a^
IL-17	4.3 ± 2.7 ^a’^	5.0 ± 2.6 ^a’^	3.7 ± 1.8 ^a’^	4.3 ± 2.6 ^a^	7.7 ± 7.1 ^a^	3.8 ± 1.8 ^a^
CCL2	10.7 ± 17.4 ^a’^	8.4 ± 4.3 ^a’^	6.8 ± 4.6 ^a’^	10.6 ± 13.3 ^a^	11.6 ± 10.2 ^a^	9.5 ± 8.5 ^a^
CCL3	1.9 ± 1.5 ^a’^	1.5 ± 1.0 ^a’^	1.6 ± 1.8 ^a’^	3.7 ± 6.1 ^a^	1.7 ± 1.4 ^a^	1.6 ± 0.9 ^a^
CCL4	9.1 ± 9.5 ^a’^	5.6 ± 8.8 ^a’^	5.0 ± 13.4 ^a’^	21.1 ± 48.8 ^a^	3.8 ± 3.3 ^a^	6.3 ± 7.6 ^a^
CCL5	32.8 ± 134.4 ^a’^	3.8 ± 1.5 ^a’^.	2.9 ± 1.4 ^a’^	10.0 ± 33.8 ^a^	4.4 ± 3.1 ^a^	3.3 ± 1.5 ^a^
CCL11	11.2 ± 19.4 ^a’^	14.6 ± 36.7 ^a’^	7.2 ± 9.6 ^a’^	6.6 ± 11.8 ^a^	24.8 ± 78.1 ^a^	11.0 ± 12.7 ^a^
CSF2	15.1 ± 13.2 ^a’^	12.1 ± 7.0 ^a’^	9.4 ± 7.1 ^a’^	13.0 ± 12.0 ^a^	21.9 ± 45.6 ^a^	9.2 ± 6.4 ^a^
CSF3	131.9 ± 156.2 ^’^	58.1 ± 129.8 ^a’,b’^	44.2 ± 81.1 ^b’^	204.6 ± 253.5 ^a^	60.5 ± 109.5 ^b^	73.4 ± 122.0 ^a^
CXCL10	1346.5 ± 3779.0 ^a’^	639.9 ± 762.5 ^a’^	309.7 ± 354.0 ^a’^	512.7 ± 1064.9 ^a^	682.6 ± 1371.7 ^a^	581.2 ± 866.2 ^a^
TNF-a	31.4 ± 55.3 ^a’^	33.1 ± 41.7 ^a’^	24.7 ± 30.2 ^a’^	31.6 ± 30.6 ^a^	56.8 ± 84.7 ^a^	31.6 ± 27.9 ^a^
IFN-Y	49.0 ± 45.9 ^a’^	73.5 ± 46.4 ^a’^	62.0 ± 55.1 ^a’^	80.4 ± 68.4 ^a^	127.4 ± 110.9 ^a^	67.3 ± 54.4 ^a^
PDGF-bb	74.4 ± 133.1 ^a’^	45.6 ± 60.0 ^a’^	33.2 ± 41.6 ^a’^	64.7 ± 136.2 ^a^	90.8 ± 249.4 ^a^	31.6 ± 34.5 ^a^
bFGF	5.1 ± 7.0 ^a’^	4.2 ± 2.5 ^a’^	3.6 ± 2.5 ^a’^	4.4 ± 3.0 ^a^	6.9 ± 12.5 ^a^	3.4 ± 1.3 ^a^
VEGF	2982.6 ± 4491.4 ^a’^	3666.5 ± 4247.7 ^a’^	3541.9 ± 4860.3 ^a’^	3883.9 ± 8847.3 ^a^	2856.4 ± 2362.8 ^a^	2623.1 ± 2070.7 ^a^

Results are mean values ± SD and are expressed in picogram per milliliter. Statistical significance within the placebo group (a’, b’ and c’) and within the probiotic group (a, b and c) is denoted with different letters (*p* < 0.05). IL = interleukin; CCL = chemokine (C-C motif); CSF = colony stimulating factor; CXCL = C-X-C motif chemokine; TNF = tumor necrosis factor; IFN = interferon; PDGF = recombinant human platelet derived growth factor; bFGF = basic fibroblast growth factor; VEGF = vascular endothelial growth factor.

## References

[1] Reid G., Bocking A. (2003). The potential for probiotics to prevent bacterial vaginosis and preterm labor. Am. J. Obstet. Gynecol..

[2] Donders G.G., Van Calsteren K., Bellen G., Reybrouck R., Van den Bosch T., Riphagen I., Van Lierde S. (2009). Predictive value for preterm birth of abnormal vaginal biota, bacterial vaginosis and aerobic vaginitis during the first trimester of pregnancy. Br. J. Obstet. Gynecol..

[3] Donati L., Di Vico A., Nucci M., Quagliozzi L., Spagnuolo T., Labianca A., Bracaglia M., Ianniello F., Caruso A., Paradisi G. (2010). Vaginal microbial flora and outcome of pregnancy. Arch. Gynecol. Obstet..

[4] McMillan A., Rulisa S., Sumarah M., Macklaim J.M., Renaud J., Bisanz J.E., Gloor G.B., Reid G. (2015). A multi-platform metabolomics approach identifies highly specific biomarkers of bacterial diversity in the vagina of pregnant and non-pregnant women. Sci. Rep..

[5] Keelan J.A., Blumenstein M., Helliwell R.J., Sato T.A., Marvin K.W., Mitchell M.D. (2003). Cytokines, prostaglandins and parturition--A review. Placenta.

[6] Challis J.R., Lockwood C.J., Myatt L., Norman J.E., Strauss J.F., Petraglia F. (2009). Inflammation and pregnancy. Reprod. Sci..

[7] Balkus J., Agnew K., Lawler R., Mitchell C., Hitti J. (2010). Effects of pregnancy and bacterial vaginosis on proinflammatory cytokine and secretory leukocyte protease inhibitor concentrations in vaginal secretions. J. Pregnancy.

[8] Holst R.M., Hagberg H., Wennerholm U.B., Skogstrand K., Thorsen P., Jacobsson B. (2011). Prediction of microbial invasion of the amniotic cavity in women with preterm labor: Analysis of multiple proteins in amniotic and cervical fluids. Br. J. Obstet. Gynecol..

[9] Nugent R.P., Krohn M.A., Hillier S.L. (1991). Reliability of diagnosing bacterial vaginosis is improved by a standardized method of gram stain interpretation. J. Clin. Microbiol..

[10] Gloor G.B., Hummelen R., Macklaim J.M., Dickson R.J., Fernandes A.D., MacPhee R., Reid G. (2010). Microbiome profiling by illumina sequencing of combinatorial sequence-tagged PCR products. PLoS ONE.

[11] Hummelen R., Fernandes A.D., Macklaim J.M., Dickson R.J., Changalucha J., Gloor G.B., Reid G. (2010). Deep sequencing of the vaginal microbiota of women with HIV. PLoS ONE.

[12] Srinivasan S., Hoffman N.G., Morgan M.T., Matsen F.A., Fiedler T.L., Hall R.W., Ross F.J., McCoy C.O., Bumgarner R., Marrazzo J.M. (2012). Bacterial communities in women with bacterial vaginosis: High resolution phylogenetic analyses reveal relationships of microbiota to clinical criteria. PLoS ONE.

[13] Romero R., Hassan S.S., Gajer P., Tarca A.L., Fadrosh D.W., Nikita L., Galuppi M., Lamont R.F., Chaemsaithong P., Miranda J. (2014). The composition and stability of the vaginal microbiota of normal pregnant women is different from that of non-pregnant women. Microbiome.

[14] Romero R., Hassan S.S., Gajer P., Tarca A.L., Fadrosh D.W., Bieda J., Chaemsaithong P., Miranda J., Chaiworapongsa T., Ravel J. (2014). The vaginal microbiota of pregnant women who subsequently have spontaneous preterm labor and delivery and those with a normal delivery at term. Microbiome.

[15] Aagaard K., Riehle K., Ma J., Segata N., Mistretta T.A., Coarfa C., Raza S., Rosenbaum S., Van den Veyver I., Milosavljevic A. (2012). A metagenomic approach to characterization of the vaginal microbiome signature in pregnancy. PLoS ONE.

[16] Chaban B., Links M.G., Jayaprakash T.P., Wagner E.C., Bourque D.K., Lohn Z., Albert A.Y., van Schalkwyk J., Reid G., Hemmingsenk S.M. (2014). Characterization of the vaginal microbiota of healthy Canadian women through the menstrual cycle. Microbiome.

[17] Verstraelen H., Vilchez-Vargas R., Desimpel F., Jauregui R., Vankeirsbilck N., Weyers S., Verhelst R., De Sutter P., Pieper D.H., Van De Wiele T. (2016). Characterisation of the human uterine microbiome in non-pregnant women through deep sequencing of the V1-2 region of the 16S rRNA gene. PeerJ.

[18] FAO/WHO (2001). Joint FAO/WHO expert consultation on evaluation of health and nutritional properties of probiotics in food. http://www.fao.org/3/a-a0512e.pdf.

[19] Homayouni A., Bastani P., Ziyadi S., Mohammad-Alizadeh-Charandabi S., Ghalibaf M., Mortazavian A.M., Mehrabany E.V. (2014). Effects of probiotics on the recurrence of bacterial vaginosis: A review. J. Low. Genit. Tract Dis..

[20] Macklaim J.M., Clemente J.C., Knight R., Gloor G.B., Reid G. (2015). Changes in vaginal microbiota following antimicrobial and probiotic therapy. Microb. Ecol. Health Dis..

[21] Reid G. (2001). Probiotic agents to protect the urogenital tract against infection. Am. J. Clin. Nutr..

[22] Reid G., Brigidi P., Burton J.P., Contractor N., Duncan S., Fargier E., Hill C., Lebeer S., Martín R., McBain A.J. (2015). Microbes central to human reproduction. Am. J. Reprod. Immunol..

[23] Walsh C.J., Guinane C.M., O’Toole P.W., Cotter P.D. (2014). Beneficial modulation of the gut microbiota. FEBS Lett..

[24] Reid G., Charbonneau D., Kochanowski B., Beuerman D., Poehner R., Bruce A.W. (2003). Oral use of Lactobacillus rhamnosus GR-1 and L. fermentum RC-14 significantly alters vaginal flora: Randomized, placebo-controlled trial in 64 healthy women. FEMS Immunol. Med. Microbiol..

[25] Yeganegi M., Watson C.S., Martins A., Kim S.O., Reid G., Challis J.R., Bocking A.D. (2009). Effect of *Lactobacillus rhamnosus* GR-1 supernatant and fetal sex on lipopolysaccharide-induced cytokine and prostaglandin-regulating enzymes in human placental trophoblast cells: Implications for treatment of bacterial vaginosis and prevention of preterm labor. Am. J. Obstet. Gynecol..

[26] Yeganegi M., Leung C.G., Martins A., Kim S.O., Reid G., Challis J.R., Bocking A.D. (2011). *Lactobacillus rhamnosus* GR-1 stimulates colony-stimulating factor 3 (granulocyte) (CSF3) output in placental trophoblast cells in a fetal sex-dependent manner. Biol. Reprod..

[27] Li W., Yang S., Kim S.O., Reid G., Challis J.R., Bocking A.D. (2014). Lipopolysaccharide-induced profiles of cytokine, chemokine, and growth factors produced by human decidual cells are altered by *Lactobacillus rhamnosus* GR-1 supernatant. Reprod. Sci..

[28] Kim S.O., Sheikh H.I., Ha S.D., Martins A., Reid G. (2006). G-CSF-mediated inhibition of JNK is a key mechanism for *Lactobacillus rhamnosus*-induced suppression of TNF production in macrophages. Cell. Microbiol..

[29] Yang S., Li W., Challis J.R., Reid G., Kim S.O., Bocking A.D. (2014). Probiotic *Lactobacillus rhamnosus* GR-1 supernatant prevents lipopolysaccharide-induced preterm birth and reduces inflammation in pregnant CD-1 mice. Am. J. Obstet. Gynecol..

[30] Aitchison J. (1986). The Statistical Analysis of Compositional Data.

[31] Gloor G.B., Wu J.R., Pawlowsky-Glahn V., Egozcue J.J. (2016). It’s all relative: Analyzing microbiome data as compositions. Ann. Epidemiol..

[32] Gloor G.B., Macklaim J.M., Vu M., Fernandes A.D. (2016). Compositional uncertainty should not be ignored in high-throughput sequencing data analysis. Aus. J. Stat..

[33] Fernandes A.D., Macklaim J.M., Linn T.G., Reid G., Gloor G.B. (2013). ANOVA- like differential expression analysis for mixed population RNA-seq. PLoS ONE.

[34] Fernandes A.D., Reid J.N., Macklaim J.M., McMurrough T.M., Edgell D.R., Gloor G.B. (2014). Unifying the analysis of high-throughput sequencing datasets: Characterizing RNA-seq, 16S rRNA gene sequencing and selective growth experiments by compositional data analysis. Microbiome.

[35] Magurran A.E. (2004). Measuring Biological Diversity.

[36] Kramer M.S., Platt R.W., Wen S.W., Joseph K.S., Allen A., Abrahamowicz M., Blondel B., Bréart G. (2001). Fetal/Infant Health Study Group of the Canadian Perinatal Surveillance System. A new and improved population-based Canadian reference for birth weight for gestational age. Pediatrics.

[37] Krauss-Silva L., Moreira M.E., Alves M.B., Braga A., Camacho K.G., Batista M.R., Almada-Horta A., Rebello M.R., Guerra F. (2011). A randomised controlled trial of probiotics for the prevention of spontaneous preterm delivery associated with bacterial vaginosis: Preliminary results. Trials.

[38] Menard J.P., Fenollar F., Henry M., Bretelle F., Raoult D. (2008). Molecular quantification of *Gardnerella vaginalis* and *Atopobium vaginae* loads to predict bacterial vaginosis. Clin. Infect. Dis..

[39] Antonio M.A., Meyn L.A., Murray P.J., Busse B., Hillier S.L. (2009). Vaginal colonization by probiotic *Lactobacillus crispatus* CTV-05 is decreased by sexual activity and endogenous lactobacilli. J. Infect. Dis..

[40] Gille C., Böer B., Marschal M., Urschitz M.S., Heinecke V., Hund V., Speidel S., Tarnow I., Mylonas I., Franz A. (2016). Effect of probiotics on vaginal health in pregnancy. EFFPRO, a randomized controlled trial. Am. J. Obstet. Gynecol..

[41] Ravel J., Gajer P., Abdo Z., Schneider G.M., Koenig S.S., McCulle S.L., Karlebach S., Gorle R., Russell J., Tacket C.O. (2011). Vaginal microbiome of reproductive-age women. Proc. Natl. Acad. Sci. USA.

[42] Reid G. (2016). Cervico-vaginal microbiomes—Threats and possibilities. Trends Endocronol. Metabol..

[43] Pramanick R., Mayadeo N., Warke H., Begum S., Aich P., Aranha C. (2019). Vaginal microbiota of asymptomatic bacterial vaginosis and vulvovaginal candidiasis: Are they different from normal microbiota?. Microb. Pathog..

[44] Walsh A.M., Crispie F., Kilcawley K., O’Sullivan O., O’Sullivan M.G., Claesson M.J., Cotter P.D. (2016). Microbial succession and flavor production in the fermented dairy beverage Kefir. mSystems.

[45] Park D.H. (2018). Effects of carbon dioxide on metabolite production and bacterial communities during kimchi fermentation. Biosci. Biotechnol. Biochem..

[46] Milani C., Alessandri G., Mancabelli L., Lugli G.A., Longhi G., Anzalone R., Viappiani A., Duranti S., Turroni F., Ossiprandi M.C. (2019). Bifidobacterial distribution across Italian cheeses produced from raw milk. Microorganisms.

[47] Menezes L.A.A., Sardaro M.L.S., Duarte R.T.D., Mazzon R.R., Neviani E., Gatti M., De Dea Lindner J. (2020). Sourdough bacterial dynamics revealed by metagenomic analysis in Brazil. Food Microbiol..

[48] Ling Z., Kong J., Liu F., Zhu H., Chen X., Wang Y., Li L., Nelson K.E., Xia Y., Xiang C. (2010). Molecular analysis of the diversity of vaginal microbiota associated with bacterial vaginosis. BMC Genomics.

[49] Costa de Freitas A., Chaban B., Bocking A., Money D.M., Hill J.E., the VOGUE Study team (2014). Characterization of the vaginal microbiome in pregnancy. Genome.

[50] Fettweis J.M., Serrano M.G., Brooks J.P., Edwards D.J., Girerd P.H., Parikh H.I., Huang B., Arodz T.J., Edupuganti L., Glascock A.L. (2019). The vaginal microbiome and preterm birth. Nat. Med..

[51] Gardiner G.E., Heinemann C., Bruce A.W., Beuerman D., Reid G. (2003). Persistence of *Lactobacillus fermentum* RC-14 and *Lactobacillus rhamnosus* GR-1 but not *L. rhamnosus* GG in the human vagina as demonstrated by randomly amplified polymorphic DNA. Clin. Diagn. Lab. Immunol..

[52] Verdenelli M.C., Cecchini C., Coman M.M., Silvi S., Orpianesi C., Coata G., Cresci A., Di Renzo G.C. (2016). Impact of probiotic SYNBIO® administered by vaginal suppositories in promoting vaginal health of apparently healthy women. Curr. Microbiol..

[53] Nenadic D.B., Pavlovic M.D. (2008). Cervical fluid cytokines in pregnant women: Relation to vaginal wet mount findings and polymorphonuclear leukocyte counts. Eur. J. Obstet. Gynecol. Reprod. Biol..

[54] Chandiramani M., Seed P.T., Orsi N.M., Ekbote U.V., Bennett P.R., Shennan A.H., Tribe R.M. (2012). Limited relationship between cervico-vaginal fluid cytokine profiles and cervical shortening in women at high risk of spontaneous preterm birth. PLoS ONE.

[55] Whitcomb B.W., Schisterman E.F., Luo X., Chegini N. (2009). Maternal serum granulocyte colony-stimulating factor levels and spontaneous preterm birth. J. Womens Health (Larchmt).

[56] Dugoua J.J., Machado M., Zhu X., Chen X., Koren G., Einarson T.R. (2009). Probiotic safety in pregnancy: A systematic review and meta-analysis of randomized controlled trials of *Lactobacillus, Bifidobacterium*, and *Saccharomyces* spp.. J. Obstet. Gynaecol. Can..

